# The Internet as a Vehicle to Communicate Health Information During a Public Health Emergency: A Survey Analysis Involving the Anthrax Scare of 2001

**DOI:** 10.2196/jmir.6.1.e8

**Published:** 2004-03-03

**Authors:** Anne F Kittler, John Hobbs, Lynn A Volk, Gary L Kreps, David W Bates

**Affiliations:** ^1^Division of General Medicine and Primary CareDepartment of MedicineBrigham and Women's Hospital(now at) Strategic Policy on Ethics and InnovationSector Policy DirectorateMinistry of HealthNew Zealand; ^2^Department of Clinical and Quality Analysis, Information SystemsPartners Healthcare System93 Worcester StreetWellesley MA 02481USA; ^3^Health Communication and Informatics Research BranchNational Cancer Institute6130 Executive Boulevard, MSC 7365Bethesda MD 20892USA

**Keywords:** bioterrorism, public health, communication, electronic mail, inequality, behavior

## Abstract

**Background:**

The recent public health risks arising from bioterrorist threats and outbreaks of infectious diseases like SARS (Severe Acute Respiratory Syndrome) highlight the challenges of effectively communicating accurate health information to an alarmed public.

**Objective:**

To evaluate use of the Internet in accessing information related to the anthrax scare in the United States in late 2001, and to strategize about the most effective use of this technology as a communication vehicle during times of public health crises.

**Methods:**

A paper-based survey to assess how individuals obtained health information relating to bioterrorism and anthrax during late 2001.We surveyed 500 randomly selected patients from two ambulatory primary care clinics affiliated with the Brigham and Women's Hospital in Boston, Massachusetts.

**Results:**

The response rate was 42%. While traditional media provided the primary source of information on anthrax and bioterrorism, 21% (95% CI, 15% - 27%) of respondents reported searching the Internet for this information during late 2001. Respondents reported trusting information from physicians the most, and information from health websites slightly more than information from any traditional media source. Over half of those searching the Internet reported changing their behavior as a result of information found online.

**Conclusions:**

Many people already look to the Internet for information during a public health crisis, and information found online can positively influence behavioral responses to such crises. However, the potential of the Internet to convey accurate health information and advice has not yet been realized. In order to enhance the effectiveness of public-health communication, physician practices could use this technology to pro-actively e-mail their patients validated information. Still, unless Internet access becomes more broadly available, its benefits will not accrue to disadvantaged populations.

## Introduction

The public may be more aware of the dangers of infectious diseases today than in any other era in recent history. The possibility of bioterrorism has become a real concern, as has preventing and controlling the spread of naturally occurring outbreaks such as the recent SARS (severe acute respiratory syndrome) epidemic. The current global environment, where travel brings diverse and distant individuals into close proximity within a matter of hours, also allows for the accidental transportation of harmful microorganisms that until relatively recently remained more geographically isolated. This context means that public health authorities and the public face a set of unique health risks, especially in a time of crisis [1]. One such crisis occurred in October 2001 when anthrax spores were spread via the US postal system, alarming a public already anxious due to the September 11^th^ terrorist attacks. Although investigation ultimately revealed that only four letters containing anthrax had entered the postal system, this relatively small-scale dispersion generated confusion and panic among the public and the media, and illustrated the challenge of communicating information about risk and reality to an alarmed public.

During the anthrax threat, the traditional media presented the public with an enormous amount of information on the emerging events, but the information provided was extremely variable, often shallow, and not always validated by health authorities. While some public health authorities endeavored to communicate evidence-based facts, other coverage simultaneously offered conflicting and often confusing accounts of what was happening, as well as varying advice on the dangers of anthrax and how to protect oneself  [2]. Such a context made it difficult for the public to decide which sources to trust and what advice to follow [3]. Research has shown that individuals view physicians as very trustworthy sources of information, [4,5,6] yet most of the information regarding anthrax that was available to the public came through sources such as television news reports and newspapers, and this information was often not backed or reviewed by physicians.

In addition to traditional media sources, Internet websites (public and private) provided a complementary source of health information. Individuals often use the Internet to validate and expand upon information they have read or heard elsewhere [7]. However, there is little information on people's searching behavior or on how they are influenced by the material they find. We hypothesized that during the anthrax scare of 2001 at least a small segment of the population benefited from Internet information on emerging events, and that this kind of information was useful in advising people about appropriate behavior. If this was the case, how can this technology be leveraged to have a broader effect? What are strategies to facilitate wider use, and to ensure public access to quality Internet information? What is the Internet's place in the public health response to an emergency such as the anthrax scare? We designed a survey to further explore these questions with the ultimate goal of understanding how the Internet could best be used as a communication vehicle during a public health crisis.

## Methods

This study was approved by the Partners HealthCare institutional review board. A survey was designed to assess how individuals obtained health information relating to bioterrorism and/or anthrax during late 2001 (Appendix 1). The survey was an original design and not based on any previously existing surveys. The survey was written in English and professionally translated into Spanish. A consent form explaining the study and any possible risks to subjects was also written in English and professionally translated into Spanish. The survey was pre-tested with a small sample of patients from an outpatient clinic at the Brigham and Women's Hospital and changes to the survey were made according to patient suggestions and reactions.

Five hundred patients were randomly selected to participate in this study. Patients were selected from the patient panels of two ambulatory primary care clinics affiliated with the Brigham and Women's Hospital in Boston, Massachusetts. Only patients who were over 18 years of age and who spoke English and/or Spanish were eligible for selection for the study. In August 2002, surveys and consent forms were mailed to the randomly selected patients. Opt-out cards were also included in the mailing so that patients could decline participation if desired. A Spanish translation of the mailing was sent to those identified as primarily Spanish speaking. In the following six months, three subsequent mailings were sent to those who did not respond to the survey. Respondents were compensated for their time and effort with a $10.00 voucher. In an effort to increase the response rate, non-respondents were telephoned after the final mailing and asked if they would be willing to complete the survey.

Descriptive analyses were performed on the survey results using SAS and Excel. Chi-squared analyses were used to compare survey respondents to the overall clinic population, and to compare those who searched the Internet for information on anthrax and bioterrorism with those who did not. Results are shown as percentages with 95% confidence intervals.

## Results

We received 209 completed surveys for a response rate of 42%. The population of survey respondents differed significantly from the overall clinic population in the demographic categories of race (p<0.001) and age (p<0.001), but not gender (p=0.08); the population of survey respondents contained a greater percentage of white and middle-aged patients than the overall clinic population. More respondents self-identified as white than as any other category (Table 1). More reported an annual household income of greater than $75,000 than any other category, although the remaining respondents were relatively evenly distributed throughout the other income categories. Just over half were college-educated. The majority of survey respondents reported good or excellent health.

**Table 1:** Demographic information on survey respondents

Demographic	Count	Percentage
**Sex**		
Female	117	56%
Male	89	43%
**Unknown**	3	1%
**Education Level**		
Did not graduate from high school	30	14%
Graduated from high school or GED	25	12%
Some college education or 2-year degree	37	18%
College degree or more	108	52%
Unknown	9	4%
**Race**		
Caucasian	137	66%
Black or African American	17	8%
Asian	9	4%
Native American or Alaskan	3	1%
Other	33	16%
Unknown	10	5%
**Annual Household Income**		
Less than $10,000	25	13%
$10,000 - $24,999	21	11%
$25,000 - $34,999	28	14%
$35,000 - $49,999	33	17%
$50,000 - $74,999	27	14%
Over $75,000	64	32%

The survey data suggest that respondents were relatively sophisticated regarding computer use. Eighty-six percent (95% CI, 81% - 91%) used computers. Ninety-six percent (95% CI, 93% - 99%) had access to the Internet, and 86% (95% CI, 81% - 91%) had been using the Internet for 2 years or more.

The 15% (95% CI, 10% - 20%) of respondents who reported that their health was "fair" or "poor" reported less computer use and access than the overall surveyed population. Thirty-eight percent (95% CI, 21% - 55%) of those in fair or poor health reported not using a computer at all. Only 63% (95% CI, 46% - 80%) of those in fair or poor health had Internet access, and only 28% (95% CI, 12% - 44%) had used the Internet for two years or more.

**Figure 1:**
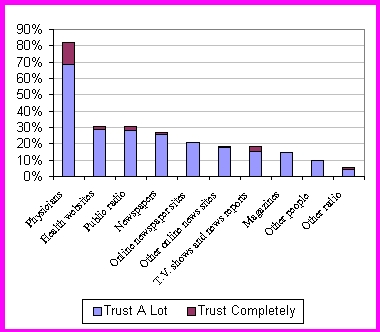
Percentage of survey respondents reporting they trust various forms of communication either "a lot" or "completely"

Respondents trusted health information from their physicians more than health information from other sources. Health websites were the next most trusted source, and were slightly more trusted than public radioand newspapers, and much more trusted than online newspaper sites, other online news sources, TV shows and news reports, magazines, other people, and other radio sources (Figure 1).

During late 2001, respondents received very little information on anthrax and/or bioterrorism from physicians, reportedly the most trusted source of information. Only 4% (95% CI, 1% - 7%) of respondents reported that their physician gave them information about anthrax in person. However, extrapolating from the proportion of patients expected to have had office visits during this time, it is possible that up to 12% of patients seeing their physicians received information about anthrax. Just 1% (95% CI, 0% - 2%) said that their physician sent them information on anthrax through the postal mail. Only one respondent (< 1%) reported receiving an e-mail from their physician with information on anthrax.

Only 12% (95% CI, 7% - 17%) reported that they obtained "a lot" of information from health websites, the next most-trusted source after physicians. Despite television shows and news reports being reported as the least trusted source of information, more people (51% (95% CI, 44% - 58%) reported receiving "a lot" of information about anthrax and bioterrorism from this source than from any other source.

Sixteen percent of respondents (95% CI, 10% - 22%) said that as a result of September 11^th^ and the anthrax scare, their overall use of the Internet increased between September and December of 2001. Twenty-one percent (95% CI, 15% - 27%) of respondents reported searching the Internet for information on bioterrorism and/or anthrax during late 2001. This group reflected a population that tended to be more educated and wealthier than the overall surveyed population, with a greater proportion of White (Table 2). Of those who searched the Internet for information on bioterrorism and/or anthrax, 54% (95% CI, 41% - 67%) reported that they did so because they wanted more information than they were getting from other sources. In addition, 63% (95% CI, 54% - 72%) of searchers turned to the Internet for information on the risk of catching anthrax and/or on how to protect oneself from anthrax exposure. Eighty-eight percent (95% CI, 79% - 97%) of searchers reported that they did not get any advice on where to search for information on anthrax and/or bioterrorism. Sixty-five percent (95% CI, 53% - 77%) located their information by searching for the word "anthrax" or "bioterrorism". Forty percent (95% CI, 27% - 53%) obtained their Internet information from online newspapers, 25% (95% CI, 14% - 36%) from private health websites like WebMD or Medscape, and 26% (95% CI, 14% - 38%) from public health websites like www.cdc.gov (Centers for Disease Control).

Those who searched the Internet tended to be white, female, and well-educated, although statistical analyses did not reveal any statistically significant differences in the demographic profiles of those who searched the Internet and those who did not. Note that seven survey respondents did not provide information regarding whether they searched the Internet for information on anthrax and bioterrorism, and these seven respondents are excluded from this table.

Internet information had an effect on the behavior of those who searched for such information. Of the respondents who reported searching online for information relating to anthrax and/or bioterrorism, 58% (95% CI, 44% - 72%) reported that they handled mail differently as a result, and 65% (95% CI, 52%-78%) reported that they washed their hands more often as a result of such information.

**Table 2:** Demographic information on survey respondents who searched the Internet for information on anthrax and bioterrorism and survey respondents who did not search the Internet for such information

	**Searchers** **(n=43)**	**Non-searchers** **(n=159)**	
Demographic	**Count**	**Percentage**	**Count**	**Percentage**	**Significant Difference?**
**Sex**					
Female	28	65%	84	53%	No, p = 0.15
Male	15	35%	72	45%	No, p = 0.22
Unknown	0	0%	3	2%	No, p = 0.36
**Race**					
Caucasian	31	72%	102	64%	No, p = 0.33
Black	3	7%	13	8%	No, p = 0.80
Asian	4	9%	5	3%	No, p = 0.08
Native American	1	2%	2	1%	No, p = 0.61
Other	4	9%	25	16%	No, p = 0.29
Unknown	0	0	12	8%	
**Education Level**					
8^th^ Grade or Less	1	2%	13	8%	No, p = 0.18
Some high school	2	5%	11	7%	No, p = 0.59
High school graduate or GED	2	5%	22	14%	No, p = 0.10
Some college or 2-year degree	11	26%	24	15%	No, p = 0.11
4-year college graduate	15	35%	33	21%	No, p = 0.05
More than 4 year college graduate	12	28%	47	29%	No, p = 0.83
Unknown	0	0	9	6%	No, p = 0.11
**Annual Household Income**					
Less than $10,000	3	7%	20	13%	No, p = 0.30
$10,000-$24,999	4	9%	15	9%	No, p = 0.98
$25,000-$34,999	6	14%	21	13%	No, p = 0.90
$35,000-$49,999	7	16%	25	16%	No, p = 0.93
$50,000-$74,999	8	19%	18	11%	No, p = 0.21
$75,000 +	14	33%	50	31%	No, p = 0.89
Unknown	1	2%	10	6%	No, p = 0.31

## Discussion

Public health crises may take the form of bioterrorist attacks or natural outbreaks. In such situations, it is important to quickly convey to an alarmed public not only accurate facts but also constructive advice that people can apply with confidence. Ensuring that individuals are connected to validated information as quickly as possible from sources that will most likely influence behavior positively is a key goal. Because messages from public health authorities are often delivered in the same context as other less credible information, the public is left to sift through a vast array of information to assess the personal risks associated with emerging events. While mass media campaigns through traditional media such as newspapers, television and radio are clearly influential in shaping individuals' responses to events, the difficulty inherent in assessing the legitimacy of these campaigns may make them less effective in positively affecting behavior. Our survey results suggest that in situations such as the anthrax threat, a portion of the population turns to the Internet to clarify what they may have learned through mass media campaigns or to get more information than was obtained through such campaigns. These people generally do not get advice about where to find the best Internet information, nor do they discuss the information they find with a healthcare provider. However, as hypothesized, the information they find can affect their behavior; a majority of those in our study who searched the Internet for information on the anthrax threat reported that the information they found lead them to wash their hands more often and handle mail differently, illustrating the potential of the Internet to help manage a public health emergency.

How can those who manage public health crises best realize the potential of the Internet as a communication channel? How can they ensure that accurate and influential information is reaching the greatest number of people as quickly as possible? Considering these questions and given the increased patient demand for e-mail communication with physicians' practices, [8] it is relevant to consider the role that physician-patient e-mail communication may play in shaping the management of public health emergencies. Our data support other research [4,5] that shows physicians are highly trusted sources of information and, as a result, it is logical to consider this trust when strategizing about how to most effectively deliver reliable information to a large population. Physician practices could e-mail their patients accurate information on public health concerns, including links to validated websites such as www.cdc.gov. This approach could use the relationship between the physician and patient to promote a flow of information that is more individualized on the public health issue at hand. Physician advice in this context is likely to be effective, as it is well understood by behavioral scientists that the most effective way to teach or persuade an individual to assess the risks and benefits of a particular course of action is through one-to-one contact [9]. While an e-mail from the practice of a physician is not one-to-one communication, it is certainly more personal than other communication devices such as television and newspaper and thus may be more influential. And with regard to Internet connectivity, almost all survey respondents reported having Internet access from somewhere, with most having it from home, suggesting that in times of public health emergencies authorities and public health officials would be able to reach a large proportion of the population via the web.

Even though the role of primary care physicians as disseminators of health information via e-mail has much potential, physicians identify a number of obstacles to its adoption, [10] such as medical liability risks, work load concerns, and a lack of reimbursement. Public health officials are in the position to distribute relevant information to primary care physicians and/or their staff, who could review such information and forward it to their patients with minimal time and effort. Given the inevitable growth in patient e-mail with health care practices, patient e-mail addresses will become available to practices making it possible to send large, all-patient e-mail distributions.

If during times of public health crises we are better able to facilitate the communication of health risk information and behavioral advice via the Internet through such mechanisms as increased physician-patient e-mail use, not all segments of the population will immediately benefit equally from this development. Those who searched the Internet for information on bioterrorism and/or anthrax during late 2001, tended to report good or excellent health, be Caucasian , and have higher education levels and higher average incomes than the overall surveyed population. Unfortunately, those reporting fair or poor health and who may be at the greatest risk of becoming ill when exposed to infectious disease or bioterrorist agents also reported lower levels of computer use and Internet access. These findings are consistent with previous research suggesting that Internet "connectivity" is directly related to income and is unequally distributed racially and ethnically [11]. Those in fair or poor health, who may be at the greatest risk of becoming ill when exposed to infectious disease or bioterrorist agents, may not have high levels of Internet connectivity. Those survey respondents who reported fair or poor health also reported lower levels of computer use and Internet access. Given this trend, it is likely that those who used the Internet as an information source during the anthrax threats did not belong to a more vulnerable group of people with underlying health issues. More research is necessary to determine how to best bridge these "digital divides" in terms of health status, race, ethnicity, and income, especially given the Internet's potential to positively influence behavior in times of public health emergencies. Those with less "connectivity" may become even more vulnerable if they are unable to access reliable information regarding how to best protect oneself during an outbreak or bioterrorist attack.

This study has several limitations. First, it was conducted with patients drawn from only two clinics in the Boston area, and the surveyed population may not be representative of the population in other areas of the US or in other countries. Importantly, the surveyed population may represent a more "wired" group than the overall US population; in our study, 96% of respondents reported Internet access, whereas only 62% of the overall US population are estimated to have Internet access [12]. Also, the survey was conducted almost a year after the anthrax scare. It is possible that respondents might have answered certain questions differently had the events surrounding the anthrax scare been more recent. Additionally, the survey had a relatively low response rate, and the population of survey respondents contained a significantly greater proportion of middle-aged Caucasians than the overall clinic population from which study participants were selected.

In conclusion, the Internet has changed the way we live, work, and communicate with each other, and it is likely to become an important part of how public health and medical professionals communicate with the public. In this context, it is important to strategize about how to best convey accurate, trustworthy, and influential information regarding health risks and advice via this communication channel. These issues are especially salient when considering times of public alarm due to bioterrorist threats or other outbreaks of infectious disease, such as the situation we recently witnessed with SARS. Although the challenge of achieving equitable Internet access for all demographic groups remains, our results suggest that information obtained from trustworthy sources on the web may be effective in positively influencing the behavior of the public during public health crises. If, in addition, public health authorities provided physician practices with validated and targeted information on emerging events that could then be e-mailed to patients, this more individualized approach could provide an additional lever with which to elicit appropriate responses from the public.

## Multimedia Appendix

Downloadable WinWord file of the English version of the survey instrument used in this study. A professional Spanish translation was mailed to patients identified as Spanish speaking:
