# Supplementary material for: The Internet as a Vehicle to Communicate Health Information During a Public Health Emergency: A Survey Analysis Involving the Anthrax Scare of 2001
Source: J Med Internet Res. 2004 Mar 3;6(1):e8. doi: 10.2196/jmir.6.1.e8 (PMC1550585; doi:10.2196/jmir.6.1.e8)
Supplement: Supplementary file 1 [file jmir_v6i1e8_app1.doc]

**First, some background questions about your health:**

1. **In general, how would you describe your own health?**

- Excellent
- Very Good
- Good
- Fair
- Poor

1. **Thinking about your overall ability to take care of your general health, how confident are you that you can:**
2. **Eat right**

- Not at all confident
- Not very confident
- Somewhat confident
- Very confident

1. **Take medicine**

- Not at all confident
- Not very confident
- Somewhat confident
- Very confident

1. **Decide when to see the doctor or health care provider**

- Not at all confident
- Not very confident
- Somewhat confident
- Very confident

**Computer related questions:**

1. **Do you use a computer anywhere? (Please check all that apply)**

- Yes, from home
- Yes, from work
- Yes, from a library
- Yes, from somewhere else (not home, work, or library). Please specify where:­­­­­­­­­­­­­­­­_____________

___________________________________________________________________________

- No **→** If “No”, Skip to Question 8

1. **Do you have access to the Internet from either home or somewhere else? (Please check all that apply)**

- Yes, from home
- Yes, from work
- Yes, from a library
- Yes, from somewhere else (not home, work, or library). Please specify where:_______________

_____________________________________________________________________________

- No **→** If “No”, Skip to Question 8

1. **How long have you been using the Internet or World Wide Web?**

- 2 years or more
- 1 year to less than 2 years
- Less than 1 year
- I have not used the Internet at all

1. **On average, how often do you search for health or wellness information using the Internet or World Wide Web?**

- Daily
- Weekly
- Monthly
- Yearly
- Almost never
- Never

1. **As a result of September 11^th^ and the anthrax threat, did your use of the Internet change during the fall of 2001 (September 11^th^ through December)?**

- Increased
- Decreased
- Stayed the same

1. **Please describe how much you generally trust information from the following sources (check the appropriate box in each row)**

**Do Not Trust No Trust Trust**

**Trust At All A little Opinion A lot Completely**

## **Physicians ** **   **

## **Other people ** **   **

## **Television shows ** **   **

**and news reports**

**Public radio **  **   **

**Other radio **  **   **

**Newspapers **  **   **

**Magazines**  ****  **   **

**Online newspaper **  **   **

**sites (like nytimes.com)**

**Other online news **  **   **

**sites (like abc.com)**

**Health websites **  **   **

In the following questions we refer to bioterrorism. When we use this term it means terrorist activities using germs or chemicals that are threats to human health.

1. **Please describe how much information on anthrax and/or bioterrorism you have gotten from the following sources (check the appropriate box in each row)**

**No A little A lot of All Don’t know**

**information information information information how much**

##

## **Physicians ** **   **

## **Other people ** **   **

## **Television shows ** **   **

**and news reports**

**Public radio **  **   **

**Other radio **  **   **

**Newspapers **  **   **

**Magazines**  ****  **   **

**Online newspaper **  **   **

**sites (like nytimes.com)**

**Other online news **  **   **

**sites (like abc.com)**

**Health websites **  **   **

1. **Did you search the Internet for information on anthrax and/or bioterrorism in the fall of 2001 (September through December)?**

- Yes
- No If “No”, Skip to question 27

######

1. **What were your main reasons for searching the Internet for information on anthrax and/or bioterrorism? (Check all that apply)**

- The Internet is my main source of information
- Wanted more information than I was getting from other sources
- Did not trust information I was getting from other sources
- Other – please specify_________________________________________________________

1. **Did you find it easy to locate information on anthrax and/or bioterrorism on the Internet?**

- Very easy to locate
- Somewhat easy to locate
- Somewhat difficult to locate
- Very difficult to locate
- No opinion

1. **Did you find Internet information on anthrax and/or bioterrorism easy to understand?**

- Very easy to understand
- Somewhat easy to understand
- Somewhat difficult to understand
- Very difficult to understand
- No opinion

1. **What kinds of information on anthrax and/or bioterrorism did you search for (check all that apply)?**

- Updates on the bioterrorist threat
- Information on the risk of catching anthrax
- Information on how to protect myself from anthrax exposure
- Other _____________________________________________________________________

**15. How would you generally rate the quality of the Internet information you found on anthrax**

**and/or bioterrorism?**

- Excellent
- Very good
- Average
- Fair
- Poor
- No opinion

1. **How would you rate the quality of the information you read on the Internet on anthrax**

**and/or bioterrorism compared to other information sources (for example, newspapers)?**

- Excellent
- Very good
- Average
- Fair
- Poor
- No opinion

1. **How often did you access Internet information on anthrax and/or bioterrorism after anthrax was first understood to be a risk (October-November, 2001)?**

- More than once daily
- Once daily
- Weekly
- Monthly
- Less than monthly
- Not at all

1. **How have you located information on anthrax and/or bioterrorism on the Internet?**

- Searched for the word “anthrax”
- Searched for the word “bioterrorism”
- Went to a particular health site and searched for “anthrax” there

(Please specify site if you can):_________________________________________________

- Other (Please specify):________________________________________________________

1. **What kind of websites has most of your information on anthrax and/or bioterrorism come from?**

- Online newspaper websites
- Private health websites (for example [www.medscape.com](http://www.medscape.com/), [www.webmd.com](http://www.webmd.com/))
- Public health websites (for example www.cdc.gov)
- Other____________________________________________________________________

1. **Did you get any advice on where to search on the Internet for information on anthrax and/or bioterrorism?**

- Yes (Please specify from whom):_______________________________________________
- No

1. **Did you have a favorite website with information on anthrax and/or bioterrorism?**

- Yes (If “yes”, name site):_______________________________________________________
- No

1. **If you had a favorite website with information on anthrax and/or bioterrorism, what made that website attractive to you? (Please check all that apply)**

- It was recommended to me
- I felt it was well researched
- I found it easy to navigate
- I found it easy to understand
- I liked the look of the website
- It had all the information I needed
- I did not have a favorite website on anthrax and/or bioterrorism
- Other ___________________________________________________________________

1. **Have you forwarded Internet information on anthrax and/or bioterrorism to other people?**

- Yes
- No

1. **How often do you now use the Internet to find information on anthrax and/or bioterrorism?**

- More than once daily
- Once daily
- Weekly
- Monthly
- Less than monthly
- Not at all

1. **Did you discuss any information on anthrax and/or bioterrorism that you read on the Internet with your physician or health care provider?**

- Yes
- No

1. **As a result of the Internet information on anthrax and bioterrorism, did you:**
2. **handle mail differently?**

- Yes
- No

1. **wash your hands more often?**

- Yes
- No

1. **change your behavior in any other way?**

- Yes (If yes, please explain how you changed your behavior):______________________

_________________________________________________________________________

- No

**27. If you did not search the Internet for information on anthrax and/or bioterrorism during the**

**fall of 2001 (September through December), did anyone else do this for you?**

- Yes
- No

1. **Has your physician or healthcare provider given you information on how to reduce the risk of contracting anthrax and/or other bioterrorist threats?**

- Yes, my physician gave me information in person
- Yes, my physician gave me information over the phone
- Yes, my physician gave me information in an email
- Yes, my physician gave me information in a postal mailing
- Yes, my physician gave me information in another way (please specify):_______________ _________________________________________________________________________
- No

1. **Has your employer given you information on how to reduce the risk of contracting anthrax and/or other bioterrorist threats?**

- Yes, my employer gave me information in person
- Yes, my employer gave me information over the phone
- Yes, my employer gave me information in an email
- Yes, my employer gave me information in a postal mailing
- Yes, in another way (please specify):_______________________________________
- No

# About you

This section is optional. It is to help us learn who is benefiting from Internet health information.

1. **What is the highest grade or level of school that you have completed?**

- 8^th^ grade or less
- Some high school, but did not graduate
- High school graduate or GED
- Some college or 2-year degree
- 4-year college graduate
- More than 4-year college degree

1. **Are you of Hispanic or Latino origin or descent?**

- Hispanic or Latino
- Not Hispanic or Latino

1. **What is your race? (You may check more than one)**

### White

- Black or African-American
- Asian
- Native Hawaiian or other Pacific Islander
- American Indian or Alaskan Indian or Alaskan Native
- Other (please print): _______________________________________________________

1. **Please indicate the number of family members that you live with (include**

**yourself):_____________**

1. **Please indicate your total household annual income:**

- Less than $10,000
- $10, 000 – $24,999
- $25, 000 – $34,999
- $35, 000 – $49,999
- $50,000 – $74,999
- Over $75,000

**Thank you for your time!**

**We sincerely appreciate your help with this survey. When we receive your completed survey, we will send you your $10 check as compensation.**

**Please mail this survey back to us in the enclosed postage-paid envelope to:**

Kenneth John Hobbs

#### Division of General Medicine

Brigham and Women's Hospital

##### 75 Francis Street

Boston, MA 02115-9950
